# Deficit of glucocorticoid‐induced leucine zipper amplifies angiotensin‐induced cardiomyocyte hypertrophy and diastolic dysfunction

**DOI:** 10.1111/jcmm.15913

**Published:** 2020-11-28

**Authors:** Donato Cappetta, Antonella De Angelis, Sara Flamini, Anna Cozzolino, Oxana Bereshchenko, Simona Ronchetti, Eleonora Cianflone, Andrea Gagliardi, Erika Ricci, Concetta Rafaniello, Francesco Rossi, Carlo Riccardi, Liberato Berrino, Stefano Bruscoli, Konrad Urbanek

**Affiliations:** ^1^ Department of Experimental Medicine University of Campania 'Luigi Vanvitelli' Naples Italy; ^2^ Department of Medicine Section of Pharmacology University of Perugia Perugia Italy; ^3^ Department of Philosophy, Social Sciences and Education University of Perugia Perugia Italy; ^4^ Department of Medical and Surgical Sciences University 'Magna Graecia' of Catanzaro Catanzaro Italy; ^5^ Department of Experimental and Clinical Medicine University 'Magna Graecia' of Catanzaro Catanzaro Italy

**Keywords:** angiotensin II, diastolic dysfunction, glucocorticoid‐induced leucine zipper, glucocorticoids, myocardial hypertrophy

## Abstract

Poor prognosis in heart failure and the lack of real breakthrough strategies validate targeting myocardial remodelling and the intracellular signalling involved in this process. So far, there are no effective strategies to counteract hypertrophy, an independent predictor of heart failure progression and death. Glucocorticoid‐induced leucine zipper (GILZ) is involved in inflammatory signalling, but its role in cardiac biology is unknown. Using GILZ‐knockout (KO) mice and an experimental model of hypertrophy and diastolic dysfunction, we addressed the role of GILZ in adverse myocardial remodelling. Infusion of angiotensin II (Ang II) resulted in myocardial dysfunction, inflammation, apoptosis, fibrosis, capillary rarefaction and hypertrophy. Interestingly, GILZ‐KO showed more evident diastolic dysfunction and aggravated hypertrophic response compared with WT after Ang II administration. Both cardiomyocyte and left ventricular hypertrophy were more pronounced in GILZ‐KO mice. On the other hand, Ang II–induced inflammatory and fibrotic phenomena, cell death and reduction in microvascular density, remained invariant between the WT and KO groups. The analysis of regulators of hypertrophic response, GATA4 and FoxP3, demonstrated an up‐regulation in WT mice infused with Ang II; conversely, such an increase did not occur in GILZ‐KO hearts. These data on myocardial response to Ang II in mice lacking GILZ indicate that this protein is a new element that can be mechanistically involved in cardiovascular pathology.

## 1 INTRODUCTION

The increasing recognition of the heterogeneity of heart failure (HF) phenotypes emphasizes the need of further elucidation of underlying mechanisms. Half of the patients with HF symptoms have normal systolic function, and the prevalence of this form of HF is on the rise at a rate of 1% per year. The growing number of HF patients is paralleled with the mounting burden of lifestyle‐related co‐morbidities, such as hypertension, obesity and diabetes.^1^, ^2^ These chronic pathologies share low‐grade chronic inflammation as a common denominator that, according to the current paradigm, drives the development of diastolic dysfunction and the progression towards HF.^3^, ^4^

Glucocorticoids (GCs), the most powerful anti‐inflammatory and immunosuppressive drugs available, are commonly used to treat chronic inflammatory and autoimmune diseases.^5^ GCs regulate numerous cellular functions in the cardiovascular system, such as cell growth, apoptosis, inflammation and regulation of vascular tone.^6^ Several studies have revealed a potential benefit of modulation of GC receptors in cardiovascular diseases.^7^ However, GCs have significant dose‐dependent toxicity and reducing GC dosing has become a major theme of basic and clinical research. Several studies have reported a key role for glucocorticoid‐induced leucine zipper (GILZ) in mediating the physiological and therapeutic effects of GCs.^8^, ^9^, ^10^ GILZ, whose expression is mainly regulated by GCs, is able to modulate cell proliferation, survival (mainly apoptosis) and differentiation.^11^, ^12^, ^13^, ^14^ So far, GILZ involvement has been described in the modulation of the same immune and inflammatory responses implicated in GC‐induced immunomodulation.^11^ Indeed, GILZ mediates thymocyte survival,^14^ inhibition of nuclear factor‐κB (NF‐κB) transcriptional activity,^15^, ^16^ inhibition of ERK‐1/2 activation and of Ras‐dependent cell proliferation and oncogenic transformation.^17^ GILZ expression is not restricted to lymphoid cells, and it has been shown to play regulatory roles in adipocytes, osteoblasts and tubular renal cells.^18^, ^19^, ^20^ GILZ is also expressed in skeletal muscle tissue,^21^ where it is involved in the regulation of myogenesis and mediates GC‐induced anti‐myogenic activity.^22^ Apart the contribution to maturational programme in foetal cardiomyocytes, the role and relevance of GILZ to cardiovascular homeostasis and myocardial remodelling are unknown.^23^, ^24^ For these reasons, we investigated the involvement of GILZ in the development of myocardial dysfunction, using a GILZ‐knockout (KO) mouse and a model of left ventricular (LV) hypertrophy, fibrosis and diastolic dysfunction that develop following the infusion of angiotensin II (Ang II).

## 2 MATERIALS AND METHODS

### 2.1 Animals

All in vivo experiments were carried out in accordance with the National Ethical Guidelines (Italian Ministry of Health; DL vo 26, 4 March 2014) and the guidelines from Directive 2010/63/EU of the European Parliament. The animals were housed in the animal facilities of the University of Perugia in a controlled environment and provided with standard rodent chow and water ad libitum. Mice bearing a floxed GILZ allele were generated and maintained on a C57Bl/6 background as previously described.^25^ GILZ‐KO mice were obtained by crossing mice bearing GILZ flox alleles with transgenic mice bearing the CMV‐CRE transgene, resulting in the deletion of GILZ in all the cells of the body.^26^

### 2.2 Experimental protocol

**Figure 1:**
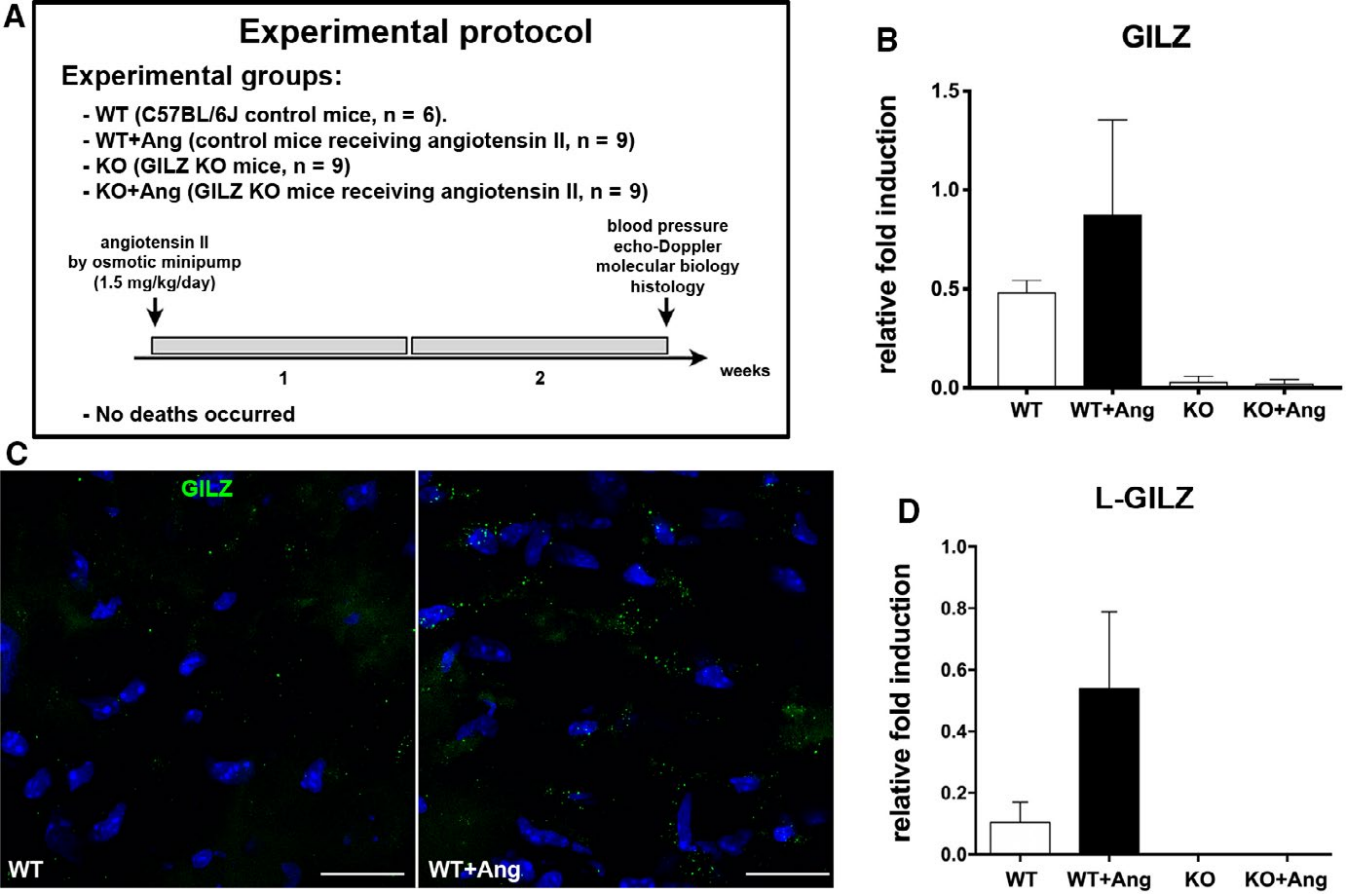
Higher GILZ mRNA expression in Ang II–infused WT mice. A, Experimental design. B, Real‐time quantitative PCR analysis of GILZ mRNA level was up‐regulated in the hearts of Ang II–treated WT mice compared with WT control mice. Data derived from 2 independent experiments and were presented relative to the expression of GAPDH mRNA. C, Representative immunofluorescent images showing higher expression of GILZ protein (green) in WT heart after Ang II administration. Nuclei were counterstained with DAPI (blue). Scale bars: 20 µm. D, Real‐time quantitative PCR analysis of L‐GILZ mRNA level was up‐regulated in the hearts of Ang II‐treated WT mice compared with WT control mice. Data derived from 2 independent experiments and were presented relative to the expression of β‐tubulin mRNA. Graphs were expressed as mean ± standard error of the mean

C57BL/6J mice were anaesthetized with 1.5% isoflurane and fitted subcutaneously beneath the scapula with a micro‐osmotic pump (ALZET, Model 1002; Durect) containing Ang II (1.5 mg/kg/d). Control mice underwent the same surgical procedure but without mini‐pump implantation. Mice were killed after 14 days of Ang II treatment. Hearts were excised and dissected, with one part being processed for histology and another snap‐frozen in liquid nitrogen for molecular analysis. Spleen was collected for the analysis of inflammatory cytokines (Figure 1A). Our protocol was based on the studies that reported that Ang II, infused for 14 days in doses comparable to that used in our work, stimulates LV hypertrophy and remodelling, along with mild cardiac dysfunction.^27^, ^28^, ^29^, ^30^

### 2.3 Blood pressure and echocardiography

After 14 days of Ang II infusion, non‐invasive measurement of blood pressure (tail cuff) was performed in conscious mice (BP‐2000; Visitech Systems). In addition, mice under anaesthesia with 1.5% isoflurane underwent transthoracic echocardiography (Vevo 770; VisualSonics). As indices of systolic function, ejection fraction and fractional shortening were calculated from the internal LV diameters in short‐axis view. Posterior wall thickness was also measured.^31^, ^32^ LV filling pressure estimation was assessed by transmitral pulsed‐wave Doppler echocardiography in the four‐chamber view. To assess diastolic function, mitral valve early wave peak (E wave), atrial wave peak (A wave), E/A ratio, isovolumetric relaxation time and E‐wave deceleration time were measured.^33^ Heart rate was recorded during blood pressure and echocardiographic monitoring.

### 2.4 Sample preparation and histological analysis

To prepare frozen sections, hearts were dissected and embedded in OCT compound (Thermo Fischer Scientific). Then, the tissue block was frozen in liquid nitrogen and stored at −80°C. Tissue sections (10 µm thick) were cut on a cryostat (CM3050 S; Leica Microsystems). For molecular biology analysis, samples were directly frozen in liquid nitrogen and stored at −80°C. Immunolabelling of GILZ (eBioscience), laminin (Sigma‐Aldrich) and FoxP3 (Thermo Fischer Scientific) was performed. Immune cells were detected by CD45 and CD3 (BD PharMingen). Myocardial fibrosis was evaluated by collagen I (Novus Biologicals) and vimentin (Abcam) stainings. Capillaries were visualized with FITC‐conjugated isolectin B4 (Vector Laboratories). Cross‐sectional microvessel profiles were counted in at least 10 fields and capillary density reported per mm^2^. Apoptotic cells were detected by terminal deoxynucleotidyl transferase–mediated UTP end labelling (TUNEL) staining using an apoptosis detection kit (Takara) according to manufacturer's instruction.^34^ Apoptotic cells were counted throughout the tissue section, and apoptotic rate was expressed per mm^2^. Myocytes were demonstrated by α‐sarcomeric actin (Sigma‐Aldrich). FITC‐ and TRITC‐conjugated secondary antibodies (Jackson ImmunoResearch) were used. Nuclei were counterstained with DAPI (Sigma‐Aldrich). Sections were analysed with confocal microscope (LSM700; Zeiss). Myocyte cross‐sectional area was measured using ImagePro Plus software (Media Cybernetics), and cell size distribution was calculated.^35^, ^36^

### 2.5 Real‐time quantitative PCR

Total RNA was extracted from the heart and spleen tissue using the RNeasy Mini Kit (Qiagen) according to the manufacturer's instructions. Conversion of total RNA to cDNA was performed using the High‐Capacity cDNA Reverse Transcription Kit (Applied Biosystems). All real‐time PCRs were performed using the 7300 Real‐Time PCR System (Applied Biosystem), and the amplifications were done using the TaqMan Gene Expression Master Mix (Applied Biosystem). GAPDH or β‐tubulin was used as an endogenous control to normalize the variability in expression levels, and results were expressed as fold increase. The qPCR TaqMan probes (Applied Biosystems) were as follows: GILZ (Tsc22d3): Mm00726417_m1; FoxP3: Mm00475162_m1; IFN‐γ: Mm01168134_m1; IL‐1β: Mm00434228_m1; TNF‐α: Mm00443258_m1; TGF‐β: Mm01178820_m1; GATA4: Mm00484689_m1; CTGF: Mm01192933_g1; and Bax: Mm0043205. Quantitative analysis of expression of L‐GILZ transcript isoform was performed using SYBR Green Gene Expression Master Mix (Applied Biosystems). Specific primers to identify L‐GILZ were the following: For: ACCGCAACATAGACCAGACC; Rev: CACAGCGTACATCAGGTGGT.

### 2.6 Western Blot

Protein extracts were obtained using RIPA buffer supplemented with protease (Sigma‐Aldrich) and phosphatase (Thermo Fisher Scientific) inhibitor cocktails. Western blot analyses were performed using antibody against total NF‐κB, phospho‐NF‐κB at serine 536 (phospho‐NF‐κB^Ser536^; Cell Signaling Technology), Caspase‐1 (Santa Cruz Biotechnology) and lamin B1 (nuclear loading control; Abcam), as previously described.^13^

### 2.7 Statistics

Echocardiographic and blood pressure data were expressed as mean ± standard deviation; PCR and Western blot results were expressed as mean ± standard error of the mean. Multiple comparisons were tested by one‐way ANOVA and Bonferroni's post‐test. Data analysis was performed using GraphPad Prism 4. *P*‐values were two‐sided and considered statistically significant if *P* < .05.

## 3 RESULTS

### 3.1 GILZ expression in the heart

Hearts of GILZ‐KO mice lacked the transcript at the baseline and upon Ang II exposure. The up‐regulation of GILZ mRNA was detected in the myocardium of Ang II–infused WT mice, suggesting the involvement of this protein in the response to Ang II (Figure 1B). In addition, immunolocalization of GILZ in Ang II–exposed myocardium of WT animals showed the up‐regulation of GILZ at protein level (Figure 1C). Interestingly, also the L‐GILZ isoform expression, present in the heart of WT mice, was up‐regulated after Ang II exposure (Figure 1D).

### 3.2 Blood pressure and heart function

**Figure 2:**
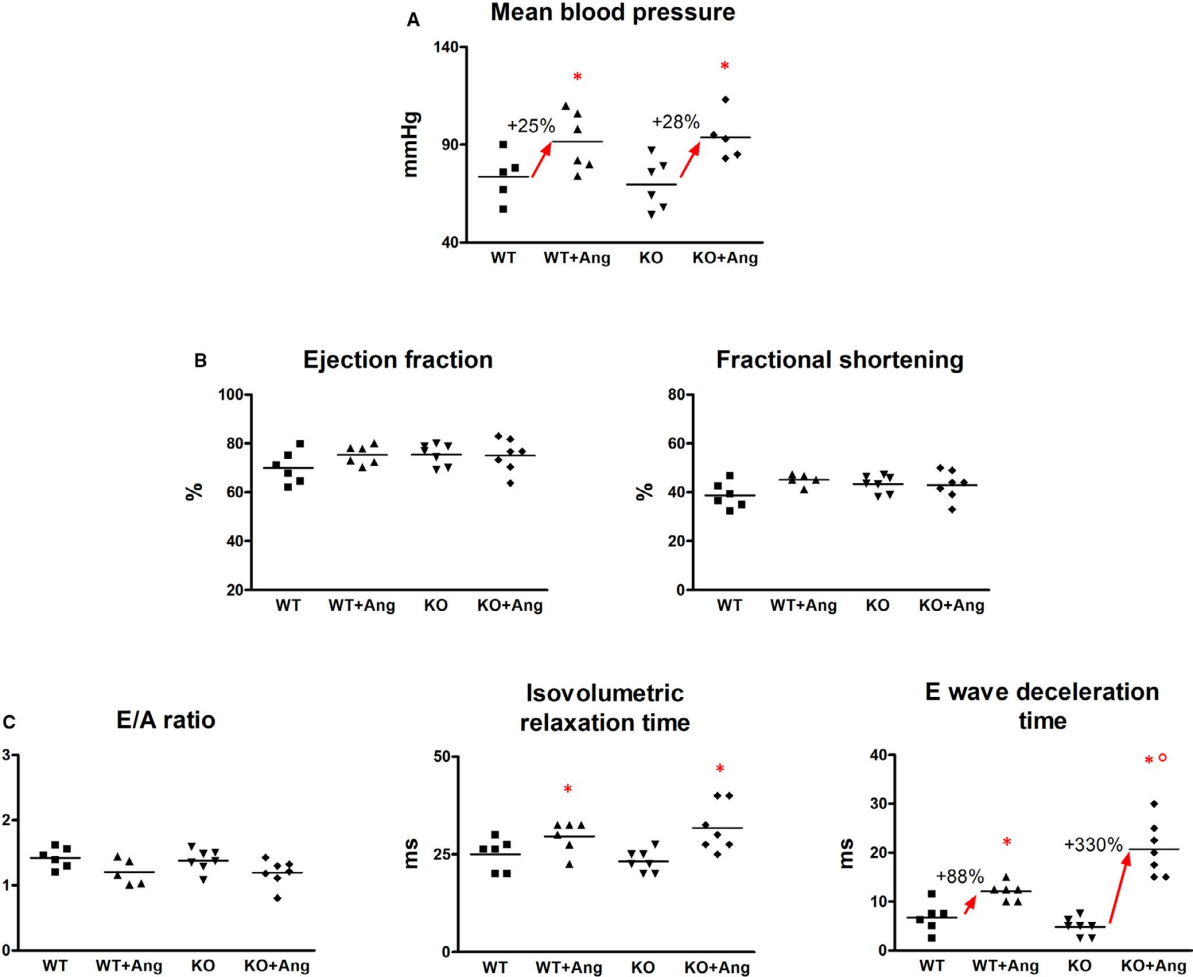
The lack of GILZ partly aggravated diastolic function after Ang II. A, Rise in mean blood pressure upon Ang II did not present any statistical difference between WT and KO mice. B, Unchanged ejection fraction and fractional shortening demonstrated unimpaired systolic function. C, Evidence of diastolic dysfunction upon infusion of Ang II; KO + Ang mice showed a higher increment of E‐wave deceleration time. *P < .05 vs strain‐matched control; °*P* < .05 vs WT + Ang

Infusion of Ang II resulted in a significant increase in blood pressure, with almost identical rise of mean pressure values in WT and GILZ‐KO mice (Figure 2A). In conscious animals, heart rate of WT (491 ± 79) and KO (499 ± 69) did not statistically change after Ang II (480 ± 78 and 492 ± 93, in WT + Ang and KO + Ang, respectively). The mice did not show symptoms of lung congestion, and systolic function was normal across experimental groups (Figure 2B). Conversely, diastolic function resulted compromised after infusion of Ang II. Notably, genetic ablation of GILZ influenced on the degree of diastolic dysfunction of the myocardium after Ang II infusion. The decline of diastolic function indicated by the abnormal transmitral flow was markedly more profound in GILZ‐KO mice, as evident by the significant increase in E‐wave deceleration time (Figure 2C). Heart rate recorded in anaesthetized mice during echocardiography was comparable among groups (442 ± 30 in WT; 449 ± 59 in WT + Ang; 453 ± 27 in KO; 447 ± 34 in KO + Ang).

### 3.3 Cardiac hypertrophy and capillary density

**Figure 3:**
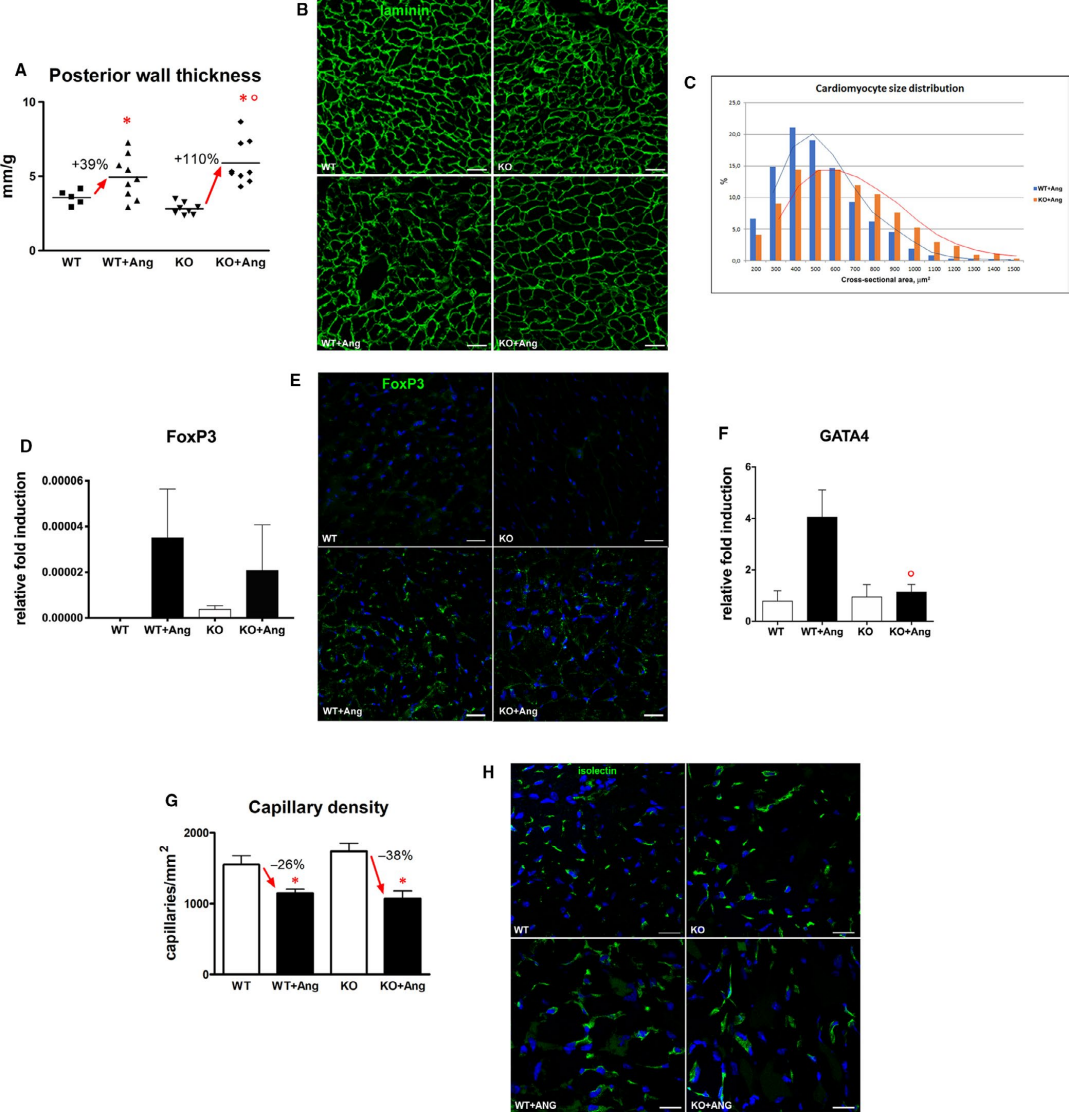
Increment of LV hypertrophy after Ang II treatment was more marked in KO animals. A, Posterior wall thickness (normalized for bodyweight) elevation was more striking in GILZ‐KO mice. B, Cardiomyocyte cross‐sectional area demonstrated by laminin immunostaining (green). C, Frequency distribution curve of myocyte cross‐sectional area was shifted rightwards in KO + Ang group in comparison with WT + Ang. D, FoxP3 transcript up‐regulation upon Ang II was less marked in GILZ‐KO hearts. E, Representative immunofluorescent images showing higher expression of FoxP3 (green) in the LV after exposure to Ang II. F, Up‐regulation of GATA4 only occurred in WT + Ang hearts. Real‐time quantitative PCR analysis of FoxP3 and GATA4 derived from 2 independent experiments and were presented relative to the expression of GAPDH mRNA. Graphs were expressed as mean ± standard error of the mean. G, Capillary rarefaction after Ang II in WT and KO hearts. H, Representative images showing lower capillary density in WT + Ang and KO + Ang mice compared with untreated animals. Endothelial cells were detected by isolectin B4 (green). Nuclei in (E, H) were counterstained with DAPI (blue). *P < .05 vs strain‐matched control; °*P* < .05 vs WT + Ang. Scale bars: (B) 50 µm, (E) 25 µm, (H) 20 µm

Echocardiographic measurement of LV wall thickness documented that Ang II infusion induced cardiac hypertrophy in both experimental groups. Notably, Ang II–induced increase in wall thickness was more prominent in GILZ‐KO hearts. Although in WT mice hypertrophy was demonstrated by 25% increase in LV wall thickness‐to‐bodyweight ratio, GILZ‐KO mice showed an enhanced hypertrophic response with an 80% increase in this parameter (Figure 3A). To evaluate hypertrophic response at the cellular level, cardiomyocyte cross‐sectional area was measured in tissue sections after visualization of base membrane with anti‐laminin antibody (Figure 3B). Frequency distribution curve of cardiomyocyte cross‐sectional area in GILZ‐KO mice was shifted towards bigger values confirming that GILZ‐KO favours a more pronounced cardiomyocyte hypertrophic reaction in response to Ang II (Figure 3C). Hypertrophic response is a complex biological process that involves several transcription factors. At this regard, the expression level of GATA4, a critical regulator of hypertrophic response, and FoxP3, a gene linking GILZ signalling to hypertrophy process, were investigated. Our findings revealed that FoxP3 transcript and protein were markedly up‐regulated after Ang II, and such an increase was less pronounced in GILZ‐KO hearts (Figure 3D,E). GATA4 mRNA expression was up‐regulated only in Ang II–infused WT mice, whereas it was not modulated in GILZ‐KO animals (Figure 3F). Evaluation of capillary density demonstrated a microvascular rarefaction in both Ang II‐treated WT and KO animals. Although no statistical differences were observed between WT + Ang and KO + Ang groups, the trend towards a lower capillary density was observed in mice lacking GILZ (KO + Ang: −38%, WT + Ang: −26%; Figure 3G,H). Collectively, these data show that GILZ may play a regulatory role in Ang II–induced cardiomyocyte hypertrophy and confirm that LV hypertrophy can represent the anatomic substrate for diastolic dysfunction.

### 3.4 Inflammatory profile

**Figure 4:**
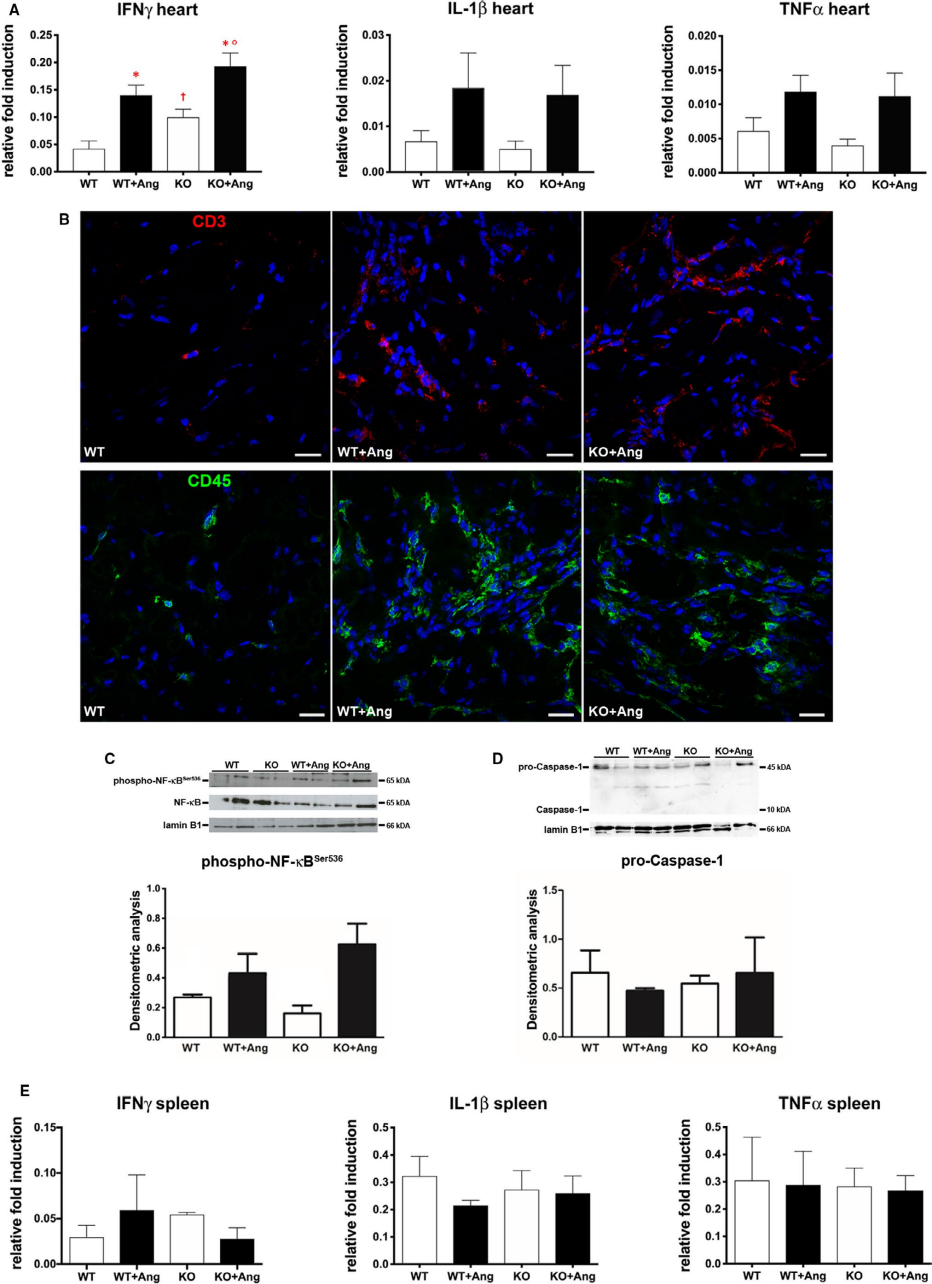
Pro‐inflammatory response in the heart enhanced by Ang II was not affected by GILZ. A, Real‐time quantitative PCR analysis of cytokine INF‐γ, IL‐1β and TNF‐α mRNA levels in the heart. B, Representative immunofluorescent images showing higher number of CD3^+^ (red, upper panels) and CD45^+^ (green, lower panels) cells in WT + Ang and KO + Ang hearts compared with WT. Nuclei were counterstained with DAPI (blue). C, Western blot analysis of total and phospho‐NF‐κB^Ser536^ expression and densitometric analysis of phospho‐NF‐κB^Ser536^/total NF‐κB. D, Western blot analysis of precursor pro–Caspase‐1 and active Caspase‐1 expression, and densitometric analysis of pro–Caspase‐1/lamin B1. Lamin B1 antibody served as loading control. Graphs were expressed as mean ± standard error of the mean. E, Real‐time quantitative PCR analysis of cytokine INF‐γ, IL‐1β and TNF‐α mRNA levels in the spleen. Data in (A, E) derived from 2 independent experiments and were presented relative to the expression of GAPDH mRNA. Graphs were expressed as mean ± standard error of the mean. *P < .05 vs strain‐matched control; °*P* < .05 vs WT + Ang; ^†^ *P* < .05 vs WT. Scale bars: 20 µm

Adverse myocardial remodelling induced by Ang II is not limited to hypertrophy. We have detected significantly modulated cardiac expression of several cytokines, consistent with the pro‐inflammatory effects of Ang II.^37^ Surprisingly, GILZ‐KO did not show differences in Ang II–induced myocardial transcripts for TNF‐α and IL‐1β with the only trend regarding IFN‐γ that may indicate higher lymphocyte activity (Figure 4A). Detection of myocardial immune cells by CD45 and CD3 immunolabelling, although clearly demonstrated enhanced infiltration in Ang II–infused mice, did not reveal significant variations between WT and GILZ‐KO (Figure 4B). Similarly, nuclear expression of phospho‐NF‐κB^Ser536^ resulted increased the following Ang II treatment but did not differ WT and GILZ‐KO mice (Figure 4C). In order to evaluate the possible activation of NLRP3 inflammasome in our experimental model, the expression of Caspase‐1, effector of inflammasome complex, was examined. Our analysis revealed that the active form of Caspase‐1 was not present in the heart of untreated and Ang II–treated WT and GILZ‐KO mice, excluding the involvement of NLRP3 inflammasome pathway (Figure 4D). To determine whether there were changes in the periphery, spleen lymphocytes were analysed. Cytokine transcripts were unchanged in all four groups, suggesting that in our experimental setting, secondary lymphoid organs were not critically involved (Figure 4E). Overall, these rather unexpected data indicate that, in our model, GILZ does not play a primary role in myocardial inflammatory process that develops upon the excess of Ang II.

### 3.5 Myocardial cell death and fibrosis

**Figure 5:**
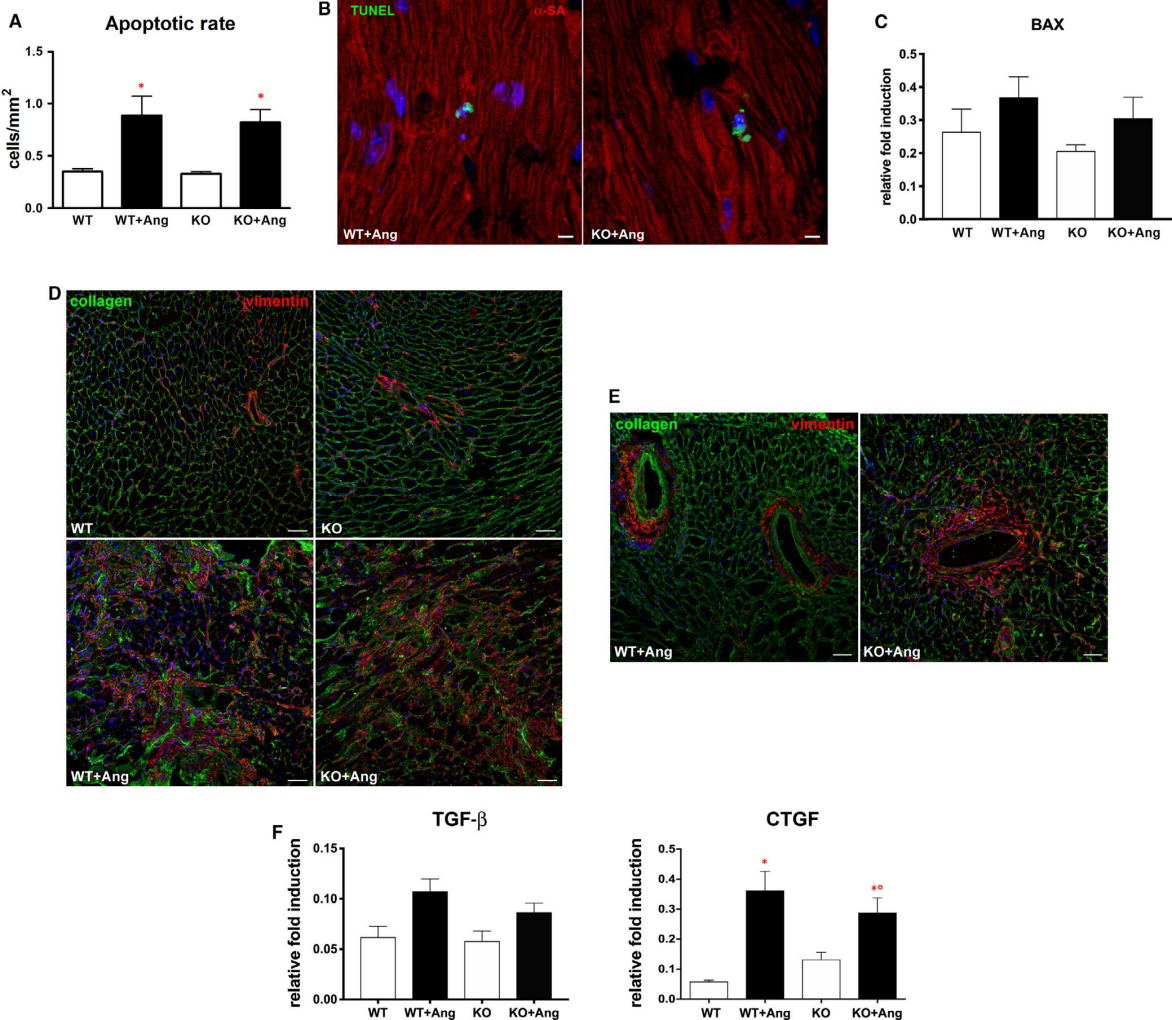
Cell death, extracellular matrix deposit and fibroblast accumulation in the heart were more extended in Ang II–exposed mice. A, Higher apoptotic rate in WT + Ang and KO + Ang hearts compared with Ang II–untreated hearts. B, Apoptotic nuclei (green) in WT + Ang and KO + Ang hearts. Cardiomyocytes were immunostained with α‐sarcomeric actin (α‐SA, red). C, Real‐time quantitative PCR analysis of BAX mRNA levels in the heart. D, Representative immunofluorescent images showing excessive accumulation of fibrillar collagen I (green) and cardiac fibroblasts (vimentin, red) in WT + Ang and KO + Ang hearts compared with Ang II‐untreated hearts. E, Examples of perivascular fibrosis in WT + Ang and KO + Ang myocardium. Nuclei in (B, D, E) were counterstained with DAPI (blue). F, Real‐time quantitative PCR analysis of TGF‐β and CTGF mRNA levels in the heart. Data in (C, F) derived from 2 independent experiments and were presented relative to the expression of GAPDH mRNA. Graphs were expressed as mean ± standard error of the mean. *P < .05 vs strain‐matched control; °*P* < .05 vs WT + Ang. Scale bars: (B) 10 µm, (D, E) 50 µm

Myocardial cell death and fibrosis, as well as inflammatory reaction, are hallmarks of adverse remodelling. Apoptotic death measured by TUNEL assay was markedly increased after Ang II, although the rate of apoptosis rose in a comparable manner in WT and KO mice (Figure 5A,B). Similarly, the transcript for pro‐apoptotic protein BAX increased after Ang II infusion, but the difference was not statistically significant (Figure 5C). In addition, following Ang II administration, extensive foci of replacement fibrosis were present in the myocardium of both WT and GILZ‐KO mice. Damaged areas were composed by the collagen deposits and fibroblasts accumulation (Figure 5D). Moreover, perivascular fibrosis that develops in parallel to focal damage was also evident (Figure 5E). However, we did not observe evident differences in the extent of damage and fibrosis between WT and GILZ‐KO hearts. This observation was confirmed by PCR analysis evidencing the overexpression of pro‐fibrotic genes, such as TGF‐β and its effector CTGF, after Ang II infusion, with no difference between WT and KO mice (Figure 5F).

## 4 DISCUSSION

The principal finding of our study was that the absence of GILZ in response to Ang II–mediated stress resulted in enhanced cardiomyocyte hypertrophy in comparison with WT hearts. In contrast, the increases in myocardial inflammatory and fibrotic reactions were similar between WT and GILZ‐KO. Because LV hypertrophy is an undeniable risk factor for HF and sudden death, and represents a valid therapeutic target, this previously unknown role of GILZ in hypertrophic growth can be of interest.^38^, ^39^

A plethora of effects mediated by Ang II has a well‐established place in cardiovascular medicine as both pathogenic component and therapeutic target.^40^ Ang II, one of the best characterized cardiovascular stressors, is the main effector of the renin‐angiotensin system, whose activation leads to hypertension, cell death, cardiac hypertrophy, inflammation, fibrosis, endothelial dysfunction and microvascular rarefaction, all major factors involved in HF pathogenesis.^41^, ^42^, ^43^ Of note, increased Ang II–induced hypertrophy in GILZ‐KO reported in the present study cannot be attributed to differences in the afterload as in both WT and GILZ‐KO, blood pressure was identically raised. This is consistent with the evidence that Ang II might exert its action on the heart (mainly through Ang II type 1 receptor), promoting fibrotic and hypertrophic pathways independently from arterial pressure changes.^44^ Our data suggest that GILZ may be involved in hypertension‐independent response to Ang II. Consistently, the importance of GC signalling in cardiovascular diseases has been demonstrated in Cushing's syndrome, in which GC excess induces adverse structural and functional cardiovascular changes that cannot be explained by the development of hypertension per se.^45^

It is well established that in addition to cardiomyocytes, also other cellular components of myocardium respond to stressors. Adverse remodelling, involving immune cells, cardiac fibroblasts and vascular cells, may manifest as inflammation‐driven endothelial dysfunction and fibrosis.^46^ In our study, the increased fibroblast accumulation and collagen deposit were evident upon Ang II infusion, but neither reactive nor replacement fibrosis was different between WT and GILZ‐KO. Although the involvement of GC signalling pathway is plausible because Ang II is among various stimuli of cortisol secretion, and GCs induce angiotensin‐converting enzyme and fibroblast activation *via* TGF‐β signalling,^47^ the role of GILZ in the response to pro‐fibrotic stimulus is unknown. Our findings of a similar fibrotic reaction in WT and KO mice do not indicate a significant involvement of GILZ in a pro‐fibrotic response to Ang II. Pathological remodelling of the myocardium is characterized not only by an increased extracellular matrix deposition, but also by a loss of cardiomyocytes and other cellular components. Herein, the continuous stress induced by Ang II infusion that mimics a persistent hyperactivity of the renin‐angiotensin system together with sustained pressure overload translated into an increased death of myocardial cells. However, in the mice lacking GILZ we did not observe any significant difference in apoptotic rate.

Detrimental effects of sterile inflammation on the myocardium are well‐recognized despite disappointing clinical results of anti‐inflammatory approach in HF. Ang II and other stressors stimulate the synthesis of pro‐inflammatory cytokines, chemokines and adhesion molecules in the heart and vessels, inducing the recruitment and activation of T and B lymphocytes, dendritic and natural killer cells.^48^, ^49^ Additionally, GILZ is a known mediator of the immune response, shown to limit inflammation by inhibiting cytokine expression in Th17 immune cells.^50^ Herein, Ang II infusion determined an up‐regulation of inflammatory genes in the heart, but unexpectedly, GILZ ablation did not affect Ang II‐driven development of myocardial inflammation. Myocardial CD45^+^ and CD3^+^ cells were increased by Ang II at a comparable degree in WT and GILZ‐KO. In addition, the activation of NLRP3 inflammasome in this context of sterile inflammation was not detected in the hearts of WT and GILZ‐KO mice. These data suggest that the pathways triggered by Ang II in cardiac fibroblasts and immune cells do not critically cross with GILZ. To date, GILZ was studied mainly as a part of an anti‐inflammatory effector of GCs,^11^, ^12^ but it may have an impact on myocardial biology given the importance of GC signalling in the heart.^24^, ^51^ Animals with a cardiomyocyte‐specific ablation of GC receptor experience an altered expression of a vast number of cardiac genes and die prematurely, developing LV hypertrophy and dysfunction.^51^ Clinically, excess of GCs in Cushing's syndrome associates with a series of cardiovascular complications, including myocardial fibrosis and hypertrophy.^52^ GILZ‐KO mice used here did not show spontaneous alteration in cardiac phenotype, but structural and functional abnormalities that became evident after Ang II administration.

Myocardial hypertrophy was the principal component that was at variance in the absence of GILZ that may explain the enhanced deterioration of diastolic function in GILZ‐KO hearts. Also, differences in diastolic performance in the presence of a similar extent of fibrosis underline the contribution of cardiomyocyte subcellular changes to LV relaxation and myocardial stiffness. This underlines the regulatory role of GILZ in the response to neurohormonal stress as a potential repressor of cell hypertrophy stimulated by Ang II. This is in line with the evidence that links hypertrophy to GC signalling.^51^, ^53^, ^54^ The proneness to hypertrophic phenotype in mice lacking GILZ has likely resulted from multifaceted molecular and cellular aspects. A mechanistic hint emerged from the Ang II–induced myocardial expression of transcription factor FoxP3. A relative reduction in FoxP3 level was present in the stressed myocardium of GILZ‐KO mice, probably reflecting a regulatory role of GILZ in the expression of FoxP3 in cardiomyocytes. This is consistent with the mechanistic link between FoxP3 and GILZ documented in immune cells.^10^ Although the details of molecular interplay of GILZ and FoxP3 in cardiomyocytes remain to be determined, our hypothesis is that the absence of GILZ may condition the expression of FoxP3, leading to excessive hypertrophic response. This is supported by a possible protective role of FoxP3 against the development of cardiac hypertrophy.^55^ GILZ ablation was also associated with the differential myocardial expression of GATA4 upon Ang II excess. GATA4 is a key regulator of postnatal heart function and stress‐induced hypertrophy of the myocardium. It is implicated in the activation of hypertrophic response in neonatal and adult cardiomyocytes by regulating numerous hypertrophic and differentiation‐related genes.^56^, ^57^, ^58^, ^59^, ^60^ In a mouse model of pressure overload, in which a diastolic dysfunction was also observed, higher level of hypertrophic genes correlated with low expression of GATA4, obtained by partial deletion of GATA4 gene.^61^ Consistently with these findings, our data showed that a more pronounced hypertrophic response occurred in GILZ‐KO mice where low levels of GATA4 were maintained after Ang II infusion. The increase in GATA4 mRNA after Ang II present only in WT mice seems to point to a regulatory role of GILZ in GATA4 expression, although the question of how the lack of GILZ may affect myocardial level of GATA4 awaits the answer. Several proteins involved in the control of cellular response to stress that physically interact with GILZ may also participate in molecular events leading to excessive hypertrophy.^11^, ^22^ In this regard, the role of NF‐κB, c‐Jun/c‐Fos heterodimer, Raf‐1, Ras and histone deacetylase 1 needs to be explored.

Because our model consists of a whole‐body gene ablation of GILZ, it is very likely that, in addition to cardiomyocytes, other myocardial cells targeted by Ang II are involved. Both in physiological and pathological conditions, coronary endothelial cells have a recognized role as dynamic regulators of surrounding cardiomyocytes, thus affecting their growth and function.^62^, ^63^, ^64^, ^65^ In this context, a large body of evidence points to the effects of Ang II and pressure overload on endothelial compartment and angiogenic profile of the myocardium.^65^, ^66^ Our analysis of capillary density revealed a significant rarefaction of coronary microvasculature after Ang II corroborating the hypothesis of the involvement of coronary endothelium.

In conclusion, we report a previously unknown role of GILZ in adverse myocardial remodelling in angiotensin‐induced model of cardiac hypertrophy and diastolic dysfunction. The enhanced myocardial response to Ang II in mice lacking GILZ, together with Ang II–induced rise in GILZ expression in wild‐type animals, indicates that this protein can be mechanistically involved in cardiovascular pathology. Our data introduce GILZ as a new player in the hypertrophic response of the myocardium, inviting future researches on GILZ as a potential pharmacological target and tool in cardiovascular medicine. This study is to be followed by further work on molecular mechanisms to clarify the role of GILZ signalling in the diseased myocardium.

## CONFLICTS OF INTEREST

The authors declare no competing or financial interests.

## AUTHOR CONTRIBUTION

**Donato Cappetta:** Conceptualization (equal); Formal analysis (equal); Writing‐original draft (equal). **Antonella De Angelis:** Conceptualization (equal); Supervision (equal). **Sara Flamini:** Data curation (supporting); Investigation (supporting). **Anna Cozzolino:** Investigation (supporting). **Oxana Bereshchenko:** Conceptualization (equal); Data curation (equal). **Simona Ronchetti:** Data curation (equal); Investigation (equal). **Eleonora Cianflone:** Data curation (supporting); Formal analysis (supporting). **Andrea Gagliardi:** Data curation (supporting). **Erika Ricci:** Data curation (supporting). **Concetta Rafaniello:** Funding acquisition (lead); Investigation (supporting). **Francesco Rossi:** Supervision (equal); Writing‐review & editing (equal). **Carlo Riccardi:** Supervision (equal); Writing‐review & editing (equal). **Liberato Berrino:** Funding acquisition (equal); Supervision (equal); Writing‐review & editing (equal). **Stefano Bruscoli:** Conceptualization (equal); Data curation (equal); Writing‐original draft (equal). **Konrad Urbanek:** Conceptualization (equal); Data curation (equal); Writing‐original draft (equal).

## Data Availability

The data sets generated during the current study are available.
